# Community-acquired pneumonia caused by Kluyvera intermedia: A rare case report

**DOI:** 10.1016/j.rmcr.2025.102194

**Published:** 2025-03-14

**Authors:** Li Xu, XiaoLong Zhu, Yi Liu

**Affiliations:** a Department of Pulmonary and Critical Care Medicine, Shandong Provincial Hospital Affiliated to Shandong First Medical University, Jinan, Shandong, 250021, China; b Department of Allergy, Department of Pulmonary and Critical Care Medicine, Shandong Provincial Hospital Affiliated to Shandong First Medical University, Jinan, Shandong, 250021, China; c Shandong Key Laboratory of Infections Respiratory Disease, Medical Science and Technology Innovation Center, Shandong First Medical University & Shandong Academy of Medical Sciences, Jinan, Shandong, 250117, China

**Keywords:** Kluyvera intermedia, Bacterial infection, Magnetic resonance imaging, Antibiotic therapy, Pneumonia

## Abstract

**Background:**

Kluyvera intermedia is a rare Gram-negative bacillus that predominantly functions as an opportunistic pathogen. Commonly found in environmental sources such as freshwater and soil, it has been infrequently reported in human infections, often leading to underrecognition in clinical practice. Because it can resemble more prevalent pathogens, accurate identification is crucial for effective management of infections.

**Case report:**

We report a case of pneumonia caused by Kluyvera intermedia in a 55-year-old male with a 10-year history of well-controlled hypertension. The patient initially presented with classic pneumonia symptoms, including fever, cough, and right-sided chest pain. Although he received empirical treatment with Cefotaxime Sodium and Levofloxacin at the local hospital, follow-up CT scans revealed further enlargement of the pulmonary lesions. To investigate the underlying etiology, the patient underwent MRI-guided lung biopsy at our hospital, and culture of the aspirated fluid identified Kluyvera intermedia as the causative pathogen. He was subsequently treated with Ceftizoxime sodium and Levonidazole Sodium Chloride Injection, resulting in significant clinical improvement. The patient was discharged after 22 days of hospitalization.

**Conclusion:**

Further accumulation and reporting of cases will enhance the recognition of the clinical characteristics of Kluyvera intermedia. These studies will not only expand our understanding of this rare pathogen but also provide a foundation for improved response strategies, thereby ensuring better management of similar infections in future clinical practice.

## Highlights

• This case report describes a rare instance of community-acquired pneumonia caused by Kluyvera intermedia, identified through MRI-guided pulmonary puncture. A Kluyvera intermedia community-acquired pneumonia case was confirmed by MRI-guided pulmonary puncture.
• The Kluyvera intermedia can cause severe lung infections even in those without obvious immunodeficiencies.
• Timely diagnosis and antibiotic therapy led to significant recovery, emphasizing the importance of early pathogen identification in atypical pneumonia cases. Timely diagnosis and antibiotic therapy led to significant recovery, stressing early pathogen identification's importance.

## 1 Introduction

Kluyvera intermedia is a relatively rare Gram-negative rod that typically acts as an opportunistic pathogen. Kluyvera is a group of Gram-negative rods discovered in 1956 by Japanese microbiologists Asai et al. [1], resembling an organism that Kluyver and van Niel predicted in 1936. It is often found in rivers and can be transmitted to humans through drinking water and food. Kluyvera grows well on common media, and its colonies resemble those of *Escherichia coli*. Only K. ascorbate, K. cryocrescens, and K. Georgiana have been isolated from human infections among the four Kluyvera species described [2]. Patients infected with Kluyvera intermedia typically exhibit clinical manifestations of pneumonia, including fever and cough, making it difficult to distinguish from pneumonia caused by more prevalent pathogens. Thus, accurately identifying Kluyvera intermedia is essential in clinical settings. This report presents a rare case of pneumonia due to Kluyvera intermedia in a patient from China, highlighting its clinical significance.

## 2 Case report

**Fig. 1:**
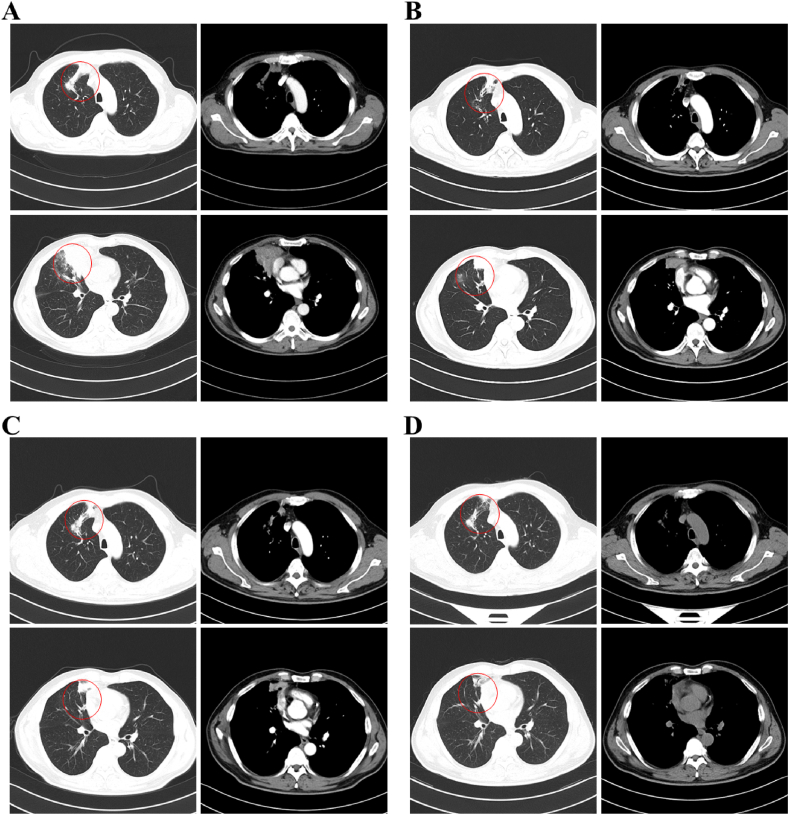
Chest CT scan before and after therapy. (A) Initial chest CT showing multiple lesions in the upper and middle lobes of the right lung, and multiple enlarged lymph nodes in the mediastinum and right hilum (indicated by red circles). (B) Chest CT after 9 days of treatment, showing reduced extent of the upper lobe lesion (indicated by red circles). (C) Chest CT after 18 days of treatment, showing further reduction in lesion extent (indicated by red circles). (D) Chest CT 1 month post-discharge, showing decreased lesion extent and less severe inflammatory changes (indicated by red circles).

**Fig. 2:**
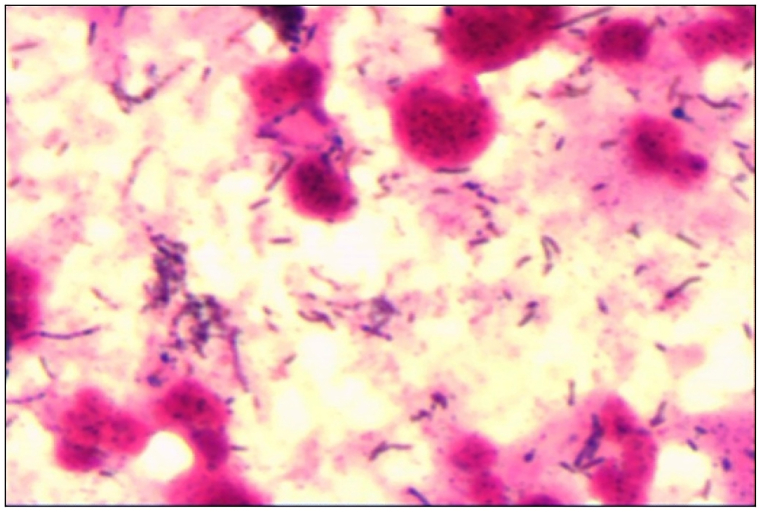
Kluyvera intermedia in a Gram-stained sample, observed under a light microscope at 10 × 100 magnification.

One month ago, a 55-year-old male presented to a local hospital with fever, cough, and right-sided chest pain, reaching a maximum temperature of 39.2 °C. Although his cough and chest pain persisted, they were alleviated by antitussive and analgesic medications. During his hospitalization, he received empirical with intravenous cefotaxime sodium (2.0 g IV drip tid) and levofloxacin (0.5 g IV drip qd), along with antitussive treatment. After one week, his chest pain and fever subsided, and his cough improved, leading to discharge two weeks later. One day prior to admission at Shandong Provincial Hospital, a follow-up chest CT scan demonstrated multiple lesions in the upper and middle lobes of the right lung, inflammation with local abscess formation, and multiple enlarged lymph nodes in the mediastinum and right hilum (Fig. 1A). He was admitted immediately. The patient had a 10-year history of hypertension, with irregular use of antihypertensive medications. His blood pressure ranged from 140 to 180 mmHg systolic to 70–110 mmHg diastolic. Upon admission, his vital signs were as follows: body temperature 36.2 °C, pulse rate 90 beats per minute, respiratory rate 22 breaths per minute, blood pressure 137/94 mmHg, and oxygen saturation of 96 % on room air. Physical examination revealed bronchial breath sounds in the right lung on auscultation. Laboratory tests revealed an elevated neutrophil count (6.63 × 10^9/L; reference range 1.8–6.3 × 10^9/L), increased C-reactive protein (50.80 mg/L; reference range 0.00–8.00 mg/L), elevated human serum amyloid A protein (97.52 mg/L; reference range 1.00–10.00 mg/L), and increased D-Dimer (0.92 mg/L; reference range 0–0.5 mg/L). The erythrocyte sedimentation rate was also elevated (80 mm/h; reference range 0–15 mm/h), and glycated hemoglobin (HbA1c) levels were increased (6.60 %; reference range 4.0–6.0 %). Liver function tests, tuberculosis antibodies, T-cell assays for *Mycobacterium tuberculosis*, G + GM tests, vasculitis antibodies, and a quantitative antinuclear antibody panel were all within normal limits. Sputum cultures, blood cultures, and comprehensive viral panels were negative. Based on the patient's symptoms and chest CT findings, we preliminarily diagnosed lung abscess and lung space-occupying lesions. Upon admission, the patient received routine empirical anti-infective therapy and cough suppression with Ceftizoxime sodium and Levonidazole sodium chloride injection. On the fourth day of hospitalization, bronchoscopy and fine-needle aspiration biopsy of the right upper lobe lesion and mediastinal lymph nodes were performed; both pathological and cytological examinations revealed no cancer cells. After excluding pulmonary malignancy, we preliminarily diagnosed the patient with a lung abscess. A repeat chest CT on day 11 revealed multiple lesions in the upper and middle lobes of the right lung with a tendency toward inflammatory lesions and local abscesses. Additionally, there was an increased number of lesions in the middle lobes and a decrease in the extent of lesions in the upper lobes, and multiple enlarged lymph nodes in the mediastinum and right hilum (Fig. 1B). Given the observed expansion and dissemination of inflammation following empirical treatment, and to further elucidate the nature of these lesions for therapy, MRI-guided pulmonary puncture was performed on day 12 of hospitalization. The aspirated fluid was subjected to bacterial culture (Fig. 2), identifying Kluyvera intermedia as the causative pathogen. Antibiotic therapy was continued to control the infection. On day 18, a repeat chest CT demonstrated a reduced extent of multiple lesions in the right upper and middle lobes, with multiple enlarged lymph nodes persisting in the right hilum and mediastinum (Fig. 1C). The patient was treated with Ceftizoxime sodium (2.0 g IV drip bid) for 22 days and Levonidazole Sodium Chloride Injection (0.5 g IV drip Q12h) for 17 days, resulting in symptomatic improvement and subsequent discharge. Upon discharge, he was prescribed Cefpodoxime Prosectil Tablets and Metronidazole Tablets for one month. A follow-up CT scan one month later in an outpatient setting demonstrated multiple enlarged lymph nodes were seen in the mediastinum and right hilum (Fig. 1D). Nonetheless, based on the follow-up results, we conclude that the anti-infective treatment effectively targeted Kluyvera intermedia, and the patient did not experience any significant adverse drug reactions throughout the course of therapy.

## 3 Discussion

In this case, the patient's clinical presentation and imaging findings initially raised suspicion of a pulmonary malignancy. However, MRI-guided pulmonary puncture and subsequent bacterial culture identified Kluyvera intermedia as the causative pathogen. After 22 days of anti-infective treatment, the patient's condition showed marked improvement, a finding rarely reported in the literature.

Kluyvera intermedia is a relatively new genus within the Enterobacteriaceae family and infrequently causes human infections. In the human body, Kluyvera species can colonize the respiratory, gastrointestinal, and urinary tracts, among other organs [3]. In previous reports, Kluyvera has predominantly been associated with infections of the gastrointestinal and urinary tract and soft tissues, often accompanied by bacteremia or even septic shock, commonly seen in urinary tract infections, cholangitis, pyelonephritis, and tenosynovitis [4,5]. Such patients often present with notable purulent discharge. Clinically, Kluyvera species are typically isolated from blood, urine, feces, tissue fluids, or sputum. In this case, however, the patient's sputum, urine, feces, and blood cultures were all negative, with imaging suggesting lung abscess-like changes. Following the exclusion of lung cancer, a bacterial culture of fluid aspirated from the lung abscess confirmed Kluyvera intermedia infection. In conclusion, percutaneous aspiration biopsy of the lung is a critical diagnostic tool for determining the nature of lung lesions. Compared with traditional imaging-guided procedures, MRI-guided biopsy provides excellent soft-tissue contrast for precise lesion localization, permits multiplanar imaging (axial, sagittal, and coronal), and reduces both the risk of complications and exposure to ionizing radiation, making it a safer and more accurate option.

Additionally, it is noteworthy that the limited case reports available on Kluyvera intermedia primarily involve opportunistic infections in immunocompromised individuals, including patients with cancer, pediatric cases, or pregnant women [[6], [7], [8]]. However, the patient in this case had no apparent history of immunodeficiency, indicating that Kluyvera intermedia can cause severe infections even in immunocompetent hosts. Although Kluyvera intermedia is not common, it is a pathogen with potential risks to humans. Consequently, early detection and treatment of Kluyvera intermedia infections are essential for a favorable prognosis. At present, the most effective in vitro agents against Kluyvera intermedia include third-generation cephalosporins, fluoroquinolones, aminoglycosides, imipenem, chloramphenicol, and nitrofurantoin. Most strains exhibit resistance to ampicillin, first-generation and second-generation cephalosporins, and ticarcillin. Other agents with varying degrees of activity include ampicillin-sulbactam, aztreonam, piperacillin, tetracycline, and trimethoprim-sulfamethoxazole [3]. This case report contributes to the understanding of Kluyvera intermedia infections leading to community-acquired pneumonia—an entity not previously described in the literature.

## 4 Conclusion

Further case accumulation and reporting will enhance understanding of the clinical characteristics of Kluyvera intermedia. Such studies will not only broaden our knowledge of this uncommon pathogen but also lay a foundation for improved treatment strategies, ultimately ensuring better management of similar infections in future clinical practice.

## CRediT authorship contribution statement

**Li Xu:** Writing – review & editing. **XiaoLong Zhu:** Writing – original draft. **Yi Liu:** Conceptualization.

## Consent to participate

Informed consent was obtained from all individual participants included in the study.

## Consent to publish

The authors affirm that human research participants provided informed consent for publication of the images in Figures and Tables.

## CARE checklist (2016) statement

The authors have read the CARE Checklist (2016), and the manuscript was prepared and revised according to the CARE Checklist (2016).

## Funding

This study was supported by the 10.13039/501100001809National Natural Science Foundation of China (82271622).

## Declaration of competing interest

The authors declare that they have no known competing financial interests or personal relationships that could have appeared to influence the work reported in this paper.
